# Low back pain among primary school teachers in Rural Kenya: Prevalence and contributing factors

**DOI:** 10.4102/phcfm.v11i1.1819

**Published:** 2019-04-17

**Authors:** Hussein E. Elias, Raymond Downing, Ann Mwangi

**Affiliations:** 1 Department of Family Medicine, College of Health Sciences, Moi University, Eldoret, Kenya; 2 Department of Behavioural Sciences, College of Health Sciences, Moi University, Eldoret, Kenya

## Abstract

**Background:**

Low back pain (LBP) has been recognised as a common occupational problem with a high prevalence among work-related musculoskeletal disorders. Although there appears to be a high prevalence of LBP among school teachers, there is inadequate information on the prevalence and predisposing factors of LBP among primary school teachers in rural Western Kenya.

**Aim:**

To determine the prevalence, factors associated with LBP and physical disability caused by LBP.

**Setting:**

The setting was public schools in rural Western Kenya selected by simple random sampling method.

**Methods:**

A cross-sectional study was conducted among primary teachers from public schools using a self-administered questionnaire. The questionnaire included information on LBP, demographic data, occupational and psychosocial factors and disability score. The 12-month prevalence, associated factors and LBP disability were analysed.

**Results:**

The 12-month self-reported prevalence of LBP among primary teachers was 64.98%, with close to 70% of them reporting minimal disability. The logistic regression analysis showed that female gender (odds ratio [OR]: 1.692, *p* < 0.02) was associated with LBP and high supervisor support (OR: 0.46, *p* < 0.003) was negatively associated with LBP.

**Conclusion:**

The prevalence of LBP among primary school teachers in rural Western Kenya is 64.98%, with the majority of them reporting minimal disability. The identified risk factors were female gender and low supervisor support. The presence of work-related psychosocial risk factors in this study suggests a comprehensive approach in evaluation and management of LBP. Preventive measures should be in place to prevent and reduce the progression of LBP disability.

**Keywords:**

low back pain; rural; Kenya; teachers, primary school; public schools; risk factors; disability.

## Introduction

Low back pain (LBP) is a muscle stiffness, tension or pain localised between the gluteal folds and the costal margin, and can appear with or without leg pain (sciatica).^1^ Typically, LBP is classified into specific and non-specific types. The non-specific LBP (NSLBP) has no identified cause and consists of the 90% of the cases of LBP. The remaining 10% of the cases have an identifiable cause such as fractures, cancer, infections and cauda equina syndrome, and therefore are known as specific LBP.^2^ Non-specific LBP is further classified into acute (less than 12 weeks) and chronic (more than 12 weeks) which is more disabling.^2^ Patients who have ever reported symptoms of LBP in the past are likely to report the same again in the future.^3^

Low back pain is a widespread problem with a lifetime prevalence of more than 70% in developed countries.^1,4,5^ In developing countries, the situation is even worse with suboptimal working conditions and the lack of ergonomic issues awareness.^6^ Predominantly, the working population both in developing and developed countries is affected, leaving a significant number of the active population disabled. The effect is similar in rural and urban regions.^7,8^ Low back pain should not be neglected as it rarely seems to be self-limiting and can present with periodic attacks with temporary remissions causing interference with daily life activities.^1,2,3,9^ The global mean prevalence of LBP regardless of period is 31% and the 12 month prevalence in Africa is 33% in adolescents and 50% in adults.^7,10^

Low back pain has been identified as one of the largest contributors to disability and therefore carries a high overall burden globally. In terms of years lived with a disability (YLDs) globally in 2016, LBP has maintained a top rank from 2010.^11^ In terms of disability adjusted life years (DALYs), it was ranked sixth globally in 2010.^8^ In high-income countries such as in North America, it was ranked first in 2010 and remains the same in 2016 and DALY was ranked third in 2010, whereas in low-income countries in the Eastern sub-Saharan Africa, YLD was ranked third in 2010 and second in 2016 and DALY was ranked 12th in 2010.^8,11^ In Kenya, the rank of YLDs following LBP has moved to the top rank in 2016 from third rank in 2010.^11,12^

Low back pain is recognised as the leading cause of work limitation, work absenteeism, early retirement and physical disability. With the peak occurring in the most reproductive age (middle age), it can cause an economic burden to an individual, community, family and government.^7,8,13^ This suggests a need for preventive measures to curb the burden and the progression of symptoms. A single preventive strategy would probably be suboptimal in reducing LBP because of many risk factors, but none are convincingly causal.^14,15^

Primary school teachers are at high risk of developing LBP because of the physically demanding nature of their daily work, which includes long hours of standing, sitting, bending and awkward posture.^16,17^ Besides teaching students, the work of teachers also includes assessing students work, preparing lessons and being involved in extracurricular activities like sports. In some areas, because of unfavourable circumstances, teachers are required to overwork in order to achieve their objectives.^16^ If the time for recovery is insufficient, pain symptoms can be triggered and lead to stress, affecting physical and mental health, which can negatively impact the performance of the teacher.^16,18^

Determining the risk factors of LBP can be a difficult task as it is recognised as a multi-factorial disorder. Some of the identified risk factors can be broadly classified into individual, occupational and psychosocial factors.^2,15^

There are limited data on the prevalence and risk factors of LBP among teachers in developing countries.^6^ The aim of this study was to analyse the prevalence and probable contributing factors of NSLBP among primary school teachers in public schools in rural Kenya.

The first objective of this study was to determine the prevalence of NSLBP among primary school teachers in Webuye Kenya. The second objective was to determine the work-related and psychosocial factors associated with LBP in primary school teachers in public schools in Webuye Kenya. The third objective was to determine the degree of disability associated with LBP in the same population.

## Research methods and design

### Study design

A cross-sectional analytical study design was used for this research.

### Setting

Randomly selected public primary schools in Webuye were used as the setting for this study. Webuye is in the western part of the country and is 309 km (geodesic distance) from the capital city of Nairobi. The region is divided into two constituencies: Webuye West and Webuye East. Webuye West covers an area of approximately 242,60 km^2^ and has a population of 129 333 people. Webuye East covers an area of approximately 161,50 km^2^ and has a population of 101 020 people. The climate is tropical with an average temperature of 24 ºC and subsistence agriculture is mainly done in the region.

### Study population and sampling strategy

Data from the sub-county education office and teachers service commission show that there are a total of 1889 teachers working in 135 public schools in Webuye. Therefore, the calculated estimate average number of teachers in each school is 14. The calculated sample size using the prevalence formula $n=\frac{Z_{1-\alpha /2}^{2}p(1-p)}{e^{2}}$ was 460 (*n*).^19^ As there were no LBP prevalence studies found in Kenya, the estimate prevalence was 50% (* p* = 0.5), margin of error was 5% (*e*) with 95% confidence interval (*Z* = 1.96) and the non-response rate used was 20%. To achieve the target population using an estimated average number of teachers in each school, the total number of schools required was 33. A list of public schools was obtained from the sub-county education office, and using a random sequence generator, the first 33 schools were randomly selected. All eligible primary teachers from the selected schools were invited to participate in the study. Teachers from primary schools, who have had back trauma or undergone surgery, with a known specific-type low back disorder, or who were physically disabled or pregnant, were excluded from the study.

### Data collection

To address the objectives of the study, the data were collected using a structured, pre-tested and self-administered questionnaire that was developed by reviewing the literature. All eligible teachers were given questionnaires and the anthropometric measurements were taken by a trained assistant or the principal researcher. The questionnaire was in English and divided into three sections. The first section was aimed to detect subjects with LBP using the validated Nordic questionnaire. The second section was aimed to detect the occupational and psychosocial risk factors using a structured questionnaire and the third section was intended to analyse the degree of disability caused by LBP using the validated Oswestry disability index questionnaire. Factors not related to work, such as physical exercise and household work, were considered as confounders and were included in the analysis and controlled for, in the multiple regression analysis.

### Data analysis

Data were entered and cleaned using Microsoft Excel and then exported to ‘R’ for analysis. Descriptive statistics were presented as frequencies, percentages and median. The associated factors in the bivariate analysis were entered in multivariate analysis to control for confounders. The variables with significant association were identified. A *p*-value of < 0.05 was considered significant.

### Ethical considerations

The study was carried out after receiving permission from the institutional research and ethics committee (approval number 0001679), sub-county education office and administrations of individual schools. Informed consent was obtained from each participant to participate in the study. Those teachers who declined to consent were not forced to participate in the study. Confidentiality was maintained. All ill teachers were referred to visit a health facility.

## Results

### Socio-demographics

A total of 417 teachers participated in the study. All the returned questionnaires by the teachers were analysed. As shown in Table 1, the majority of respondents (61%) were women. The median age of all the participants was 41 years (interquartile range [IQR] 35–49), with a median body mass index (BMI) of 24.9 (IQR 22.0–27.8). The median years of working as a teacher and the median number of hours spent at work per week were 13 years (IQR 7–24) and 40 h (IQR 40–40), respectively.

**TABLE 1:** Descriptive statistics.

Variable	Frequency (%)	Median (IQR)
Male	164.0	39.4
Female	252.0	60.6
Age in years	41.0	35.0–49.0
Years of working	13.0	7.0–24.0
Hours of work per week	40.0	40.0–40.0
BMI	24.9	22.0–27.8

BMI, body mass index; IQR, interquartile range.

### Low back pain prevalence

The self-reported prevalence of NSLBP among primary teachers in Webuye for the previous 12 months was 64.98% (*n* = 271), with a 95% confidence interval (CI) (60.39–69.58). Table 2 shows the characteristics of those who reported NSLBP. The majority of teachers with LBP did not seek health care (77.0%), nor were they hospitalised (84.6%) for the pain. More than 50.0% of teachers had their leisure and daily work activities affected and less than one-eighth changed their job as a result of back pain. Absenteeism for less than a week was seen in about half of the teachers with back pain, whereas a much smaller percentage were absent for more than a month. Half of those who experienced LBP (51.5%) had suffered the pain in the last 1 week.

**TABLE 2:** Characteristics of those with low back pain.

Variable	Frequency	Percentage
**Hospitalised**		
Yes	42	15.4
No	230	84.6
**Changed job**		
Yes	34	12.5
No	238	87.5
**Work activity**		
Yes	165	61.1
No	105	38.9
**Leisure activity**		
Yes	142	52.8
No	127	47.2
**Prevent work**		
Never	58	21.6
1–7 days	123	45.9
8–30 days	49	18.3
More than 30 days	38	14.2
**Seen doctor in past 12 months**		
Yes	62	23.0
No	207	77.0
**Pain in last 1 week**	
Yes	137	51.5
No	129	48.5

Note: All the returned questionnaires were analysed and because the questionnaires were self-administered, some questions were not answered.

Table 3 shows the characteristics of occupational and psychosocial factors known to contribute to LBP. From the data, it is evident that the majority of the teachers sit for less than 3 h but stand for more than 3 h during their time at work. More than half of the respondents found the back support of the chairs they used to be uncomfortable. Of the total respondents, 68% were happy with their jobs, with more than half reporting poor supervisor support but good co-worker support. Physical exercise is performed by 260 (62.4%) respondents, of which only a quarter exercise for more than 5 h per week.

**TABLE 3:** Characteristics of occupational and psychosocial factors known to contribute to low back pain.

Variable	Frequency	Percentage
**Hours sitting per day (*n* = 417)**		
Less than 1 h	114	27.3
1–3 h	174	41.7
3–5 h	74	17.7
More than 5 h	55	13.2
**Hours standing per day (*n* = 416)**		
Less than 1 h	6	1.4
1–3 h	40	9.6
3–5 h	125	30
More than 5 h	245	58.9
**Same chair (*n* = 416**)			
Less than 1 year	39	9.4
1–3 years	71	17.1
3–5 years	60	14.4
More than 5 years	246	59.1
**Comfortable back support of chair (*n* = 408)**	
Yes	115	28.2
No	293	71.8
**Job satisfaction (*n* = 407)**	
Yes	277	68.1
No	130	31.9
**Supervisor support (*n* = 399)**	
Low	216	54.1
High	183	45.9
**Co-worker support (*n* = 403)**	
Low	163	40.4
High	240	59.6
**Physical exercise (*n* = 417)**	
Yes	260	62.4
No	157	37.6
**Hours of exercise per week^†^ (*n* = 256)**		
More than 5 h	66	25.8
Less than 5 h	190	74.2
**Household work per week (*n* = 414)**		
More than 20 h	135	32.6
Less than 20 h	279	67.4

h, hour.

† , Among those with physical exercise.

### Risk factors of low back pain

In the bivariate analysis (* p* < 0.038), and in the final adjusted model (* p* < 0.02), gender was found to be a statistically significant variable, with the female gender associated with LBP. Although not associated with LBP, the median age, BMI, hours of work and years of work were similar in both the groups with and without LBP. Among the occupational and psychosocial factors, the statistically significant variables were supervisor support, co-worker support and physical exercise in the bivariate analysis, and in the multivariate analysis only supervisor support was significant. Low support from the supervisors was associated with LBP. Although not significant in the multivariate analysis, exercising had lower odds of developing LBP in the bivariate analysis.

**TABLE 4:** Factors associated with low back pain: Bivariate analysis.

Variable	Yes		No		*p*
	Median	IQR	Median	IQR	
**Sex**								
Male	97.00	59.1	67.00	40.9	0.0380
Female	174.00	69.0	78.00	31.0	
**Age**	41.00	35.0–50.0	42.00	35.0–48.0	0.8368
**Years of working**	13.50	8.0–24.5	12.00	7.0–23.0	0.2344
**Hours in a week**	40.00	40.0–40	40.00	40.0–45.0	0.3813
**BMI**	25.10	22.0–28.0	24.35	21.9–27.1	0.2254
**Hours sitting**							
Less than 1 h	75.00	65.8	39.00	34.2	0.8450
1–3 h	110.00	63.2	64.00	36.8	
3–5 h	51.00	68.9	23.00	31.1	
More than 5 h	35.00	63.6	20.00	36.4	
**Hours standing**							
Less than 1 h	6.00	100.0	0.00	0.0	0.3000
1–3 h	24.00	60.0	16.00	40.0	
3–5 h	81.00	64.8	44.00	35.2	
More than 5 h	159.00	64.9	86.00	35.1	
**Same chair**							
Less than 1 year	29.00	74.4	10.00	25.6	0.1850
1–3 years	39.00	54.9	32.00	45.1	
3–5 years	39.00	65.0	21.00	35.0	
More than 5 years	163.00	66.3	83.00	33.7	
**Back support**							
Yes	67.00	58.3	48.00	41.7	0.0760
No	198.00	67.6	95.00	32.4	
**Job satisfaction**							
Yes	175.00	63.2	102.00	36.8	0.2980
No	89.00	68.5	41.00	31.5	
**Supervisor support**							
Low	159.00	73.6	57.00	26.4	**0.0000**
High	102.00	55.7	81.00	44.3	
**Co-worker support**							
Low	115.00	70.6	48.00	29.4	**0.0450**
High	146.00	60.8	94.00	39.2	
**Physical exercise**							
Yes	159.00	61.2	101.00	38.8	**0.0350**
No	112.00	71.3	45.00	28.7	
**Hours of exercise**^†^							
More than 5 h	34.00	51.5	32.00	48.5	0.1250
Less than 5 h	122.00	64.2	68.00	35.8	
**Household work**							
More than 20 h	92.00	68.1	43.00	31.9	0.3840
Less than 20 h	178.00	63.8	101.00	36.2	

Note: Bold values indicate significant variables with *p* < 0.05.

h, hour; IQR, interquartile range; BMI, body mass index.

† , Among those with physical exercise.

### Disability

**FIGURE 1:**
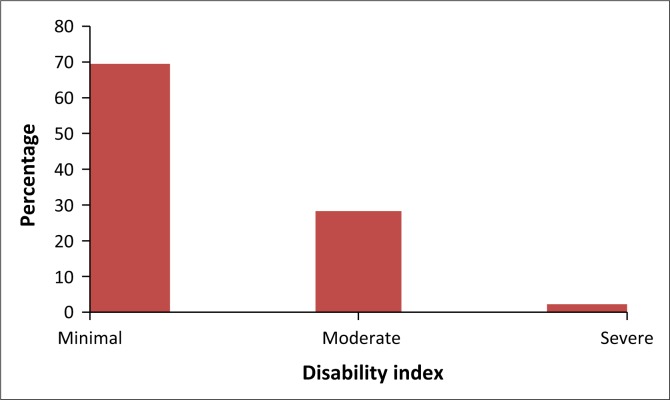
Percentage of disability index.

As shown in Figure 1, the majority of the teachers with LBP reported minimal disability. Almost a quarter of the teachers reported moderate disability, while a relatively low proportion of teachers reported severe disability.

## Discussion

### Prevalence of low back pain

The first objective of this study was to estimate the 12-month prevalence of self-reported LBP among primary teachers in Webuye. The 12-month self-reported prevalence of LBP estimated in the current study is 64.9%. This falls within the estimated prevalence of 14% – 72% among the working group in Africa.^10^ This is similar to the estimated prevalence of studies from Saudi Arabia among female teachers.^20,21^ The prevalence reported in this study was higher than the estimate from similar studies from other countries: Malaysia (40.4%), Brazil (41.4%), China (45.6%) and Germany (38.7%).^17,18,22,23^ Compared to similar studies from Africa, this study estimated a slightly higher prevalence than Botswana (55.7%) and Ethiopia (53.8%) but was less than Tanzania (82.9%).^6,24,25^ The possible reason for the difference in the prevalence could be the study participants. This study was only based on primary teachers from government schools, whereas the other studies from other countries were conducted on teachers from different levels of school (from primary to higher institution levels) and from both government and private schools. The other possible reasons for the prevalence of difference could be the difference in the facility provided for the teachers or social and economic differences between the countries.

**TABLE 5:** Risk factors associated with low back pain: Multivariate logistic regression results.

Variable	Odds ratio	*p*	95% CI
Age	1.004	0.744	0.981–1.028
**Sex**			
Male	1.000	-	-
Female	1.692	0.020	1.087–2.632
**Back support**			
Yes	1.000	-	-
No	1.507	0.091	0.937–2.425
**Supervisor support**			
Low	1.000	-	-
High	0.467	0.003	0.283–0.771
**Co-worker support**			
Low	1.000	-	-
High	0.918	0.748	0.546–1.545
**Physical exercise**			
Yes	1.000	-	-
No	1.447	0.116	0.913–2.293

Note: Bold values indicate significant variables with *p* < 0.05.

CI, confidence interval.

### Low back pain and risk factors

The second objective of this study was to determine the associated factors to LBP among teachers in Webuye. The analysis revealed interesting associations between gender and supervisor support.

In this study, female teachers were 1.5 times more likely to experience LBP. This result was consistent with that of studies conducted in the neighbouring countries such as Botswana, Ethiopia and Tanzania and in distant countries such as Malaysia and Brazil.^6,17,18,24,25^ The reason for the gender difference could be that women have a lower threshold to pain than men; therefore, women were more likely to report a pain problem than men.^6,24^ Other possible reasons for female teachers to have a higher prevalence of pain is thier lower physical strength, pregnancy, menstruation and pain related to osteoporosis.^26^ In this study, surprisingly, there was no significant association between the age and BMI with LBP. The association of BMI with LBP was also found in studies conducted in Botswana, Tanzania and Saudi Arabia.^6,21,25^ This was unlike the study in China which found an association of LBP with BMI which was more than 28 kg/m^2^ and a study in Qatar with a BMI of more than 30 kg/m^2^.^22,27^ In this study, the median BMI of those with LBP was 25.1 kg/m^2^, which when compared to the mean BMI from studies in Botswana (26.65 kg/m^2^) and Saudi Arabia (27.6 kg/m^2^) was found to be lesser, but was higher than Tanzania (22.58 kg/m^2^).^6,21,25^ The no association found in this study could possibly be because the median BMI was just above the healthy and normal range classified by the World Health Organization (18.99 kg/m^2^ – 24.99 kg/m^2^).^28^ This shows that the primary school teachers in Webuye are not in the obese category. With studies showing that the prevalence of LBP increases with increase in age and the peak occurs at 35–55 years of age,^7,15^ this study showed that no significant association with age was evident despite the median age (41 years) to be within the peak range. Similarly, this was found in a study from China where age was not significantly associated with LBP, while their mean age was 32.21 years (standard deviation [SD] 10.61 years).^22^ In the Botswana study, the mean age of the participants was 38.5 years (SD 8.62), while teachers between 40 and 50 years were 1.5 times more likely to report LBP. Similarly, in the studies in Ethiopia, the mean age was 38 years (SD 11.00) and teachers between 30 and 40 years were 1.7 times more likely to report LBP, and with the increase in age of more than 40 years, the likelihood was 2.34 times more.^6,24^ Further studies are needed to understand this interesting finding.

Sitting for more than 3 h could be a risk factor for LBP.^22^ There was a significant association between sitting for more than half an hour and standing for more than 1 h, and LBP, in a Qatar study.^27^ Similarly, prolonged sitting, standing and uncomfortable furniture was positively associated with LBP among teachers in China.^22^ An association of uncomfortable furniture with LBP was also seen in teachers in Brazil.^18^ This study did not find any significant association between LBP and the occupational factors including prolonged standing, prolonged sitting and use of uncomfortable furniture. It has been proposed that prolonged sitting itself does not increase the risk of LBP. The increase in likelihood of LBP is the combination of whole body vibration and/or awkward postures leading to loading and twisting of the spine with prolonged sitting.^22^ Another possible explanation could be obesity combined with poor posture or leading to poor posture or with an uncomfortable chair in other studies, that leads to back pain. Further studies are required to analyse the postures associated with LBP of teachers in Webuye. In the bivariate analysis, this study shows that regular physical exercise negatively associates with LBP but on further multiple regressions, it was not significant. Performing regular physical exercise has shown a negative association with LBP.^6,22,24^

Psychosocial factors play a vital role in the development of LBP and are also important in the transition from acute to chronic LBP.^29,30^ In this study, the only psychosocial factor that was significantly associated with LBP was a lack of supervisor support. High supervisor support was associated with low odds of having LBP. This was inconsistent with the study from Botswana and Ethiopia, which found that supervisor support had no association with the development of LBP.^6,24^ Similarly, a Malaysian study found no association of LBP with supervisor support but that it had a significant association with neck and shoulder pain.^29^ Other psychosocial factors, such as low co-worker support and job dissatisfaction, were not associated with LBP in this study. This was similar in the study in Botswana.^6^ In this study, the presence of psychosocial risk factors was not assessed using any tool or scoring method but was purely self-reported and subjective. More studies might be required to evaluate the psychosocial aspects of LBP. The difference in associations could be explained by the different methods used to evaluate the psychosocial factors.

The findings of this study suggest that NSLBP is partly a work-related psychosocial problem, and its management needs a more comprehensive approach. Therefore, the management interventions should also target the work-related psychosocial factors.^30^

### Disability

The majority of teachers in this study were found to have minimal disability. This is defined by the ability to cope with most living activities, and usually no treatment is indicated apart from advice on lifting, sitting and exercise. This may imply that probably the majority of teachers had pain at tolerable levels. Similarities were drawn from a study in Botswana, which showed that two-thirds of the teachers had minimal disability and none were being bedridden.^6^ This was inconsistent with the study from Tanzania, showing that the majority of teachers had severe disability (67%).^25^

Although the reported disability was minimal in this study, strategies should be in place to minimise the progression of disability from minimal to significant. This should also aim at reducing the level of pain for those with moderate or severe disability to minimal disability.

### Limitations

There are limitations identified in this study. Because it is a cross-sectional study, only associations can be made without inferences of causality. There is a possibility of recall bias and self-reporting of LBP. It is not clear whether the respondents correctly remember the presence of LBP in the past 1 year, which could lead to under- or overestimation. The presence of LBP solely depends on the self-report of the participants and is not based on an objectively verified diagnosis. Because of the large number of independent variables, there could be an underestimation of the role of the risk factors assessed within the regression analysis. Psychosocial factors were not evaluated using a scoring tool but were assessed purely subjectively, which can lead to an overestimation or underestimation.

## Conclusion

The 12-month prevalence of NSLBP among primary teachers in Webuye in this study was 64.98%. Female gender and low supervisor support were identified as the significant risk factors in the logistic regression analysis. This suggests that the condition is a partly work-related psychosocial problem as well. The majority of teachers had minimal disability; therefore, they were able to continue with their work and were not required to visit the health care facility. Further studies are required to evaluate the psychosocial and occupational risk factors associated with LBP.
